# Brain-Derived Neurotrophic Factor Loaded PS80 PBCA Nanocarrier for In Vitro Neural Differentiation of Mouse Induced Pluripotent Stem Cells

**DOI:** 10.3390/ijms18030663

**Published:** 2017-03-19

**Authors:** Chiu-Yen Chung, Martin Hsiu-Chu Lin, I-Neng Lee, Tsong-Hai Lee, Ming-Hsueh Lee, Jen-Tsung Yang

**Affiliations:** 1 Department of Neurosurgery, Chang Gung Memorial Hospital, Chia-Yi 61363, Taiwan; yen5103106@gmail.com (C.-Y.C.); martin.lin@uclmail.net (M.H.-C.L.); ming2072@cgmh.org.tw (M.-H.L.); 2 Department of Medical Research, Chang Gung Memorial Hospital, Chia-Yi 61363, Taiwan; geneasylum@yahoo.com.tw; 3 College of Medicine, Chang Gung University, Tao-Yuan 33302, Taiwan; thlee@adm.cgmh.org.tw; 4 Stroke Center and Department of Neurology, Chang Gung Memorial Hospital, Linkou 33305, Taiwan

**Keywords:** brain-derived neurotrophic factor, polybutylcyanoacrylate nanocarrier, hypoxia-responsive element, induced pluripotent stem cell

## Abstract

Brain derived neurotrophic factor (BDNF) can induce neural differentiation in stem cells and has the potential for repair of the nervous system. In this study, a polysorbate 80-coated polybutylcyanoacrylate nanocarrier (PS80 PBCA NC) was constructed to deliver plasmid DNAs (*p*DNAs) containing BDNF gene attached to a hypoxia-responsive element (HRE-cmvBDNF). The hypoxia-sensing mechanism of BDNF expression and inductiveness of the nano-formulation on mouse induced pluripotent stem cells (iPSCs) to differentiate into neurons following hypoxia was tested in vitro with immunofluorescent staining and Western blotting. The HRE-cmvBDNF appeared to adsorb onto the surface of PS80 PBCA NC, with a resultant mean diameter of 92.6 ± 1.0 nm and zeta potential of −14.1 ± 1.1 mV. HIF-1α level in iPSCs was significantly higher in hypoxia, which resulted in a 51% greater BDNF expression when transfected with PS80 PBCA NC/HRE-cmvBDNF than those without hypoxia. TrkB and phospho-Akt were also elevated which correlated with neural differentiation. The findings suggest that PS80 PBCA NC too can be endocytosed to serve as an efficient vector for genes coupled to the HRE in hypoxia-sensitive cells, and activation of the PI3/Akt pathway in iPSCs by BDNF is capable of neural lineage specification.

## 1. Introduction

Brain-derived neurotrophic factor (BDNF) belongs to the neutrophin family of growth factors that plays a crucial role in the survival, differentiation and synaptic plasticity of neurons in the mammalian nervous system [1]. In the brain, BDNF protein is mainly distributed in the olfactory bulb, cortex and hippocampal formation, and it is found at the synaptic terminals where cell-to-cell communication occur [2,3]. The release of BDNF into the extracellular space may be triggered by various patterns of neural electrical activity as well as neurotransmitters and related substances [4], and the effects are mediated via activation of the tropomysin-related kinase B (TrkB) receptor-signaling pathways such as the Ras/extracellular signal regulated kinase (ERK), phosphatidylinositol-3-OH kinase (PI3K)/Akt kinase and phospholipase C-γ 1 (PLC-γ 1) pathways [5]. The neuroprotective and pro-neurogenesic effects of BDNF have been widely explored against a myriad of neurological disorders in which reversal of the reduced BDNF level in the affected brain regions have produced encouraging results.

Several methods have been devised to enhance the regional function of BDNF [6]. The use of carrier-free recombinant BDNF represents the most simplistic method attempted; in a phase III clinical trial of amyotrophic lateral sclerosis patients, the survival benefit seen only in patients on high-dose subcutaneous BDNF reflects on the problems associated with its short half-life and limited blood–brain barrier (BBB) permeability [7], and despite the feasibility of intrathecal BDNF, evidence on clinical efficacy is lacking [8,9]. Direct and intraventricular BDNF administrations have produced positive results in animal studies [10,11]; however, the invasive nature makes these routes of administration less applicable clinically. Various moieties have been conjugated to the BDNF for added functionality: for example, the intraperitoneal administration of the fusion-peptide in rodent models of Alzheimer’s disease that consisted of BDNF and an human immunodeficiency virus (HIV)-encoded transactivator of transcription cell-penetrating peptide designed for greater BBB penetration led to a significant spatial memory improvement [12]; and, similarly, BDNF conjugated with 2000DA polyethylene glycol and OX26 monoclonal antibody to the transferrin receptor was able to reduce systemic BDNF clearance by 67%, and target the BBB to decrease the infarct volume by up to 65% when it is given intravenously to rats subjected to middle cerebral artery occlusion [13,14]. Peptidomemtics, small molecules or prodrugs that exhibit comparably better pharmacokinetics, may augment the BDNF-signaling pathway; one highlighted example for non-invasive clinical application is 7,8-dihydroxyflavone, which is a potent small molecule TrkB receptor agonist one-tenth the size of BDNF. 7,8-dihydroxyflavone has a 10 times longer circulation half-life than BDNF and readily crosses the BBB with no apparent systemic toxicity [15,16]. It lacks affinity to the p75 receptor, thus only pathways responsible for neuroprotection, plasticity and neurogenesis are activated with no effect on the apoptosis pathway [17]. Gene therapy to alter BDNF expression has been reported with the use of adeno-associated viral vectors [18,19], and an ex vivo approach with the transplantation of genetically modified stem cells to persistently express BDNF [20], however safety and the lack of site-specific and adjustable BDNF function remains problematic [21].

Nanotechnology-based delivery system can potentially overcome the pharmacokinetic barriers associated with conventional therapeutics through properties such as better encapsulation for greater bioavailability and lower toxicity, and surface modifiability with specific ligands for added functionalities such as controlled-release and targeted-delivery [22,23,24]. Nanocarriers (NCs) also exhibit favorable characteristics for penetration through the BBB, which are independent of the properties of the enclosed cargo; thus, the ability of NCs to deliver compounds that are normally excluded by the BBB such as high molecular weight and hydrophilic species is of relevance to central nervous system (CNS) therapeutics [25,26].

Polybutylcyanoacrylate nanocarrier (PBCA NC) is a synthetic polymeric colloidal drug delivery system that is known for its biocompatible, biodegradable and non-toxic properties [27,28]. PBCA NC can be synthesized under conditions that preserve the biological activity of the cargo, either by polymerization of *n*-butylcyanoacrylate (BCA) monomers or by nanoprecipitation of pre-synthesized PBCA polymers. Depending on the method of preparation, it can be made into nanospheres, in which the cargo is adsorbed on the polymer matrix, or into nanocapsules, in which the cargo is solubilized in a liquid core within a polymer shell [29]. Degradation of PBCA NC occurs by esterase-mediated hydrolysis of the ester side-chains followed by polymer loss with solvation of the degraded residue or complete depolymerization of the hydrolyzed chains [30,31]. PBCA NC has been shown to facilitate passage of relatively BBB-impermeable agents into the CNS [32]; the unmodified PBCA NC can increase the passage of BBB-impermeable hydrophilic cargoes by 20-fold across the bovin brain microvascular endothelial monolayer [33], and surface decoration with polysorbate 80 (PS80) can further enhance CNS delivery [34,35]. The anionic PBCA NC has been used as a non-viral gene delivery vector for neutrophin-3 (NT-3), which could carry up to 90% of the added DNA load and resulted a significant increase in NT-3 expression [36]. PBCA NC differs from the commonly used cationic lipoplexes, the latter are able to interact electrostatically with the negatively charged DNA to form densely packed multi-compartmental structures with optimal loading capacity, and be endocytosed by cells efficiently [37,38,39]. By contrast, PBCA NC-DNA polyplex may be less well internalized due to its negative charge, furthermore, surface adsorption of the DNA under weak non-electrostatic force may result in a lower carrying capacity and predispose the exposed and less condensed DNA to degradation, however, the DNA may unpack and dissociate more readily from the PBCA NC once it has reached the cytosol to compensate for the aforementioned barriers to gene delivery [40].

The brain is vulnerable and sensitive to alterations in oxygen supply [41], during brain injury, a reduction in brain tissue oxygen and energy failure leads to anoxic depolarization of neurons, which triggers injury cascades from the excessive release of neurotransmitters [42,43], thus, brain hypoxia could be exploited for targeted therapy. The oxygen-sensing mechanism is mediated by the hypoxia-inducible factor 1 (HIF-1), which consists of an oxygen-regulated α-subunit and a constitutionally expressed β-subunit [44]. Normoxia leads to post-translational proline hydroxylation of HIF-1α and protease degradation upon ubiquitination, whereas in hypoxia, the lack of hydroxylation stabilizes HIF-1α and enables the formation HIF-1 complex with HIF-1β [45,46], the HIF-1 complex leads to expression of hypoxia-inducible genes through its interaction with the hypoxia-responsive element (HRE) of enhancers or promoters [47].

**Scheme 1:**
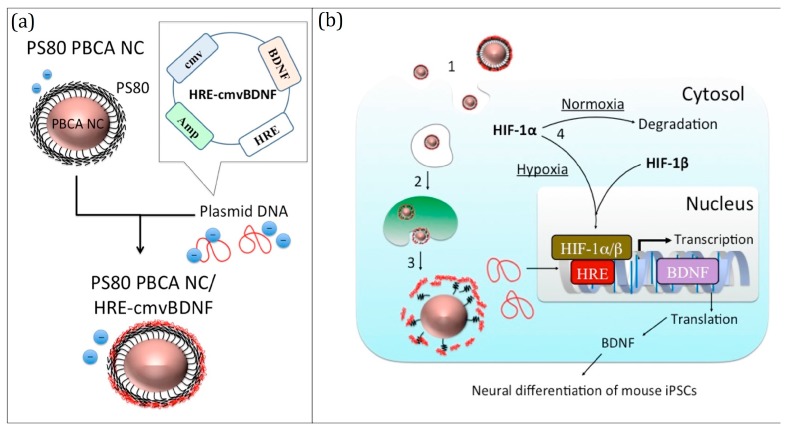
(**a**) The constructed anionic polyplex hypoxia-sensing gene delivery system, derived from mixing of polysorbate 80-coated polybutylcyanoacrylate nanocarrier (PS80-coated PBCA NC) with plasmids containing brain-derived neurotrophic factor (BDNF) gene linked to a cytomegalovirus (CMV) promotor and hypoxia-responsive element (HRE) (PS80 PBCA NC/HRE-cmvBDNF); and (**b**) the intracellular fate and hypoxia-sensing mechanism of transfection by the PS80 PBCA NC/HRE-cmvBDNF for BDNF expression. (1) The anionic polyplex is endocytosed; and (2) forms an endolysosome; (3) The NC is able to escape from the endolysosome and protect the plasmid DNA from degradation, and the weak non-electrostatic interaction between the plasmid DNA (*p*DNA) and NC facilitates unloading of the *p*DNA; (4) The transcription process (hypoxia-sensing mechanism) is augmented by the HRE; under normoxia, hypoxia-inducible factor-1α (HIF-1α) is destined for degradation, whereas, in hypoxia, HIF-1 complex is formed, which interacts with the HRE to trigger transcription of the BDNF gene.

In this study, we constructed an anionic polyplex hypoxia-sensing gene delivery system with PS80-coated PBCA NC adsorbing plasmids containing BDNF gene linked to a cytomegalovirus (CMV) promoter and an HRE (PS80 PBCA NC/HRE-cmvBDNF). The PS80 PBCA NC/HRE-cmvBDNF was characterized; the BDNF expression in response to hypoxia was tested in vitro using mouse induced pluripotent stem cells (iPSCs), and the transfection capability was determined by the BDNF-induced neural differentiation of iPSCs following a brief exposure to hypoxia. The PS80 PBCA NC/HRE-cmvBDNF construct and hypoxia-sensing mechanism of transfection are outlined in Scheme 1.

## 2. Results

### 2.1. Characterization of PS80 PBCA NC and PS80 PBCA NC/HRE-cmvBDNF

The particle size and zeta potential of PS80 PBCA NC and PS80 PBCA NC/HRE-cmvBDNF measured by Zetasizer Nano ZS90 (Malvern, Worcestershire, UK) are shown in Table 1. The mean diameter of PS80 PBCA NC was measured at 88.3 nm, which increased to 92.6 nm upon the addition of HRE-cmvBDNF. The change to the zeta potential of PS80 PBCA NC was more pronounced with the addition of HRE-cmvBDNF, which shifted from −5.3 mV to −14.1 mV, suggesting that HRE-cmvBDNF was associated with the PS80 PBCA NC by surface adsorption of the negatively charged *p*DNA.

**Figure 1:**
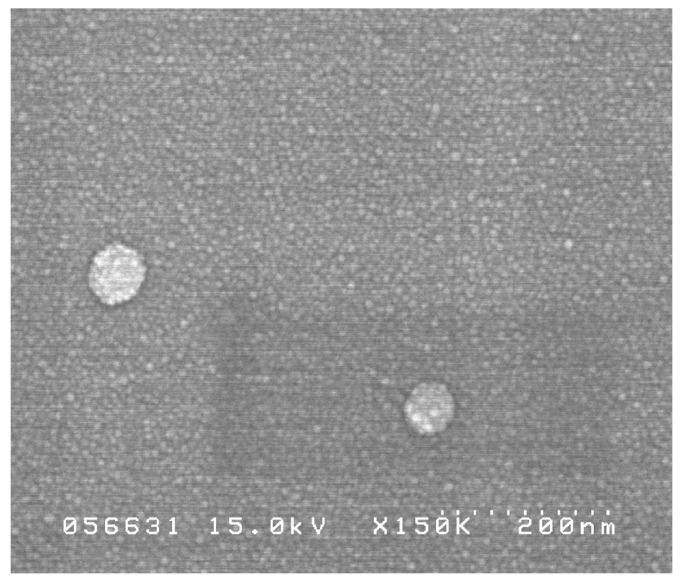
Field emission scanning electron microscope (FE-SEM) image of PS80 PBCA NC, ×150,000.

Under the field emission scanning electron microscope (FE-SEM), the PS80 PBCA NC appeared spherical and uniform in size (Figure 1), which is compatible with the results obtained by Zetasizer Nano ZS90.

**Figure 2:**
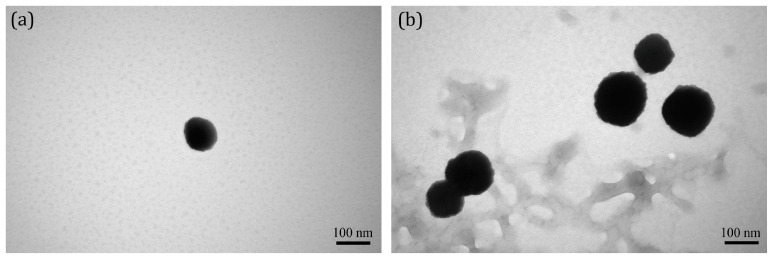
Transmission electron microscope (TEM) images of: (**a**) PS80 PBCA NC; and (**b**) PS80 PBCA NC/HRE-cmvBDNF, ×200,000.

The transmission electron microscope (TEM) images of PS80 PBCA NC appeared smooth in texture, which became more irregular and granular upon condensation of the HRE-cmvBDNF onto its surface (Figure 2).

### 2.2. pDNA Adsorption Efficiency of PS80 PBCA NC

**Figure 3:**
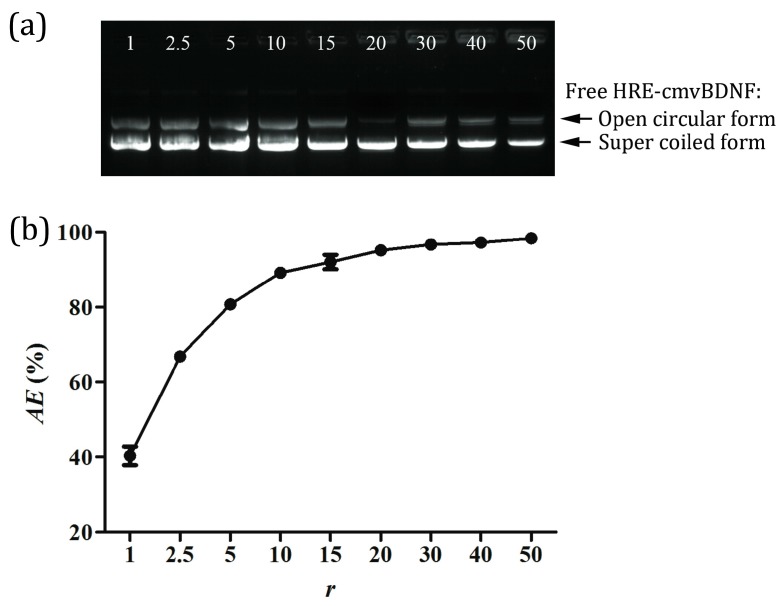
(**a**) Electrophoretic mobility analysis of PS80 PBCA NC/HRE-cmvBDNF by 0.8% agarose gel; a reduction in free HRE-cmvBDNF was observed as the weight ratio increased; and (**b**) adsorption efficiency (*AE*) of HRE-cmvBDNF on PS80 PBCA NC; an *AE* greater than 90% was reached at a weight ratio of 15:1, no significant gain in *AE* was found at higher weight ratios. *r* is the weight ratio of the PS80 PBCA NC to 2 μg/mL of HRE-cmvBDNF. *n* = 3; *p* < 0.001.

The electrophoretic mobility analysis of PS80 PBCA NC/HRE-cmvBDNF on 0.8% agarose gel and *p*DNA adsorption efficiency (*AE*) at different weight ratios of PS80 PBCA NC to 2 μg/mL of HRE-cmvBDNF are shown in Figure 3. The gel electrophoretic intensity of unbound *p*DNA obtained after centrifugation of the suspended PS80 PBCA NC/HRE-cmvBDNF indicated that PS80 PBCA NC took out free *p*DNA in a proportionate manner. Adsorption efficiency in excess of 90% was calculated with a weight ratio of 15:1, which plateaued with further increases in PS80 PBCA NC concentration.

### 2.3. Morphology and Identification of the iPSCs

**Figure 4:**
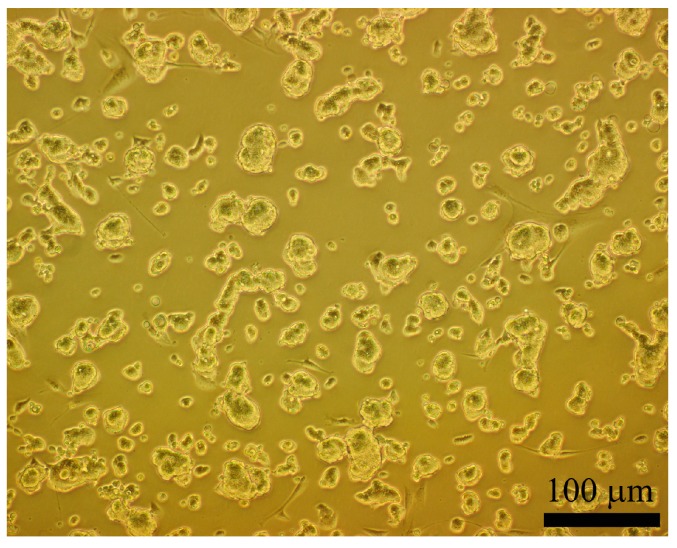
The Optical image of induced pluripotent stem cells (iPSCs) at passage-12. The iPSCs appeared as discrete clusters of variable sizes. The cell boarders were indistinct and difficult to resolve.

The morphology of iPSCs at passage-12 appeared as discrete colonies (Figure 4); the cell culture was well maintained, as spindle and ellipsoid-shaped cells representing a deviation from the original iPSCs were few in number.

**Figure 5:**
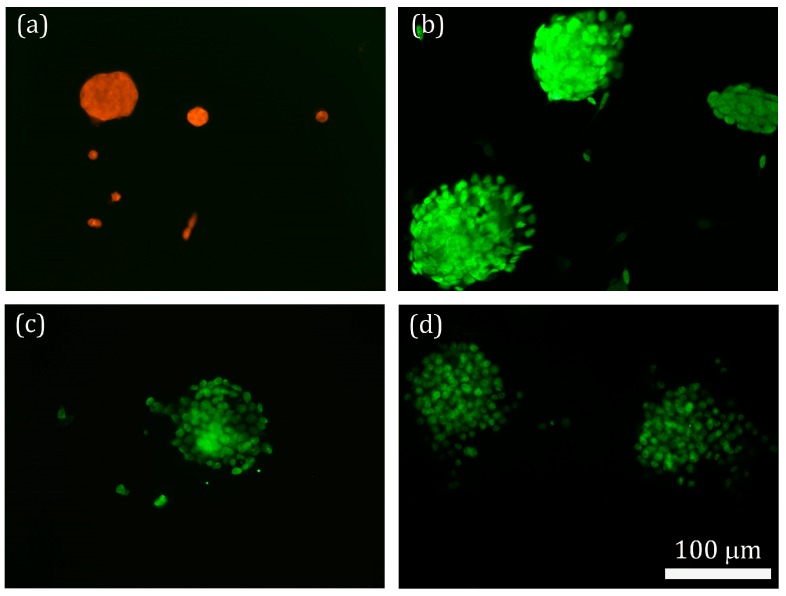
iPSCs were identified by immunofluorescent staining for: stage-specific mouse embryonic antigen (SSEA-1) (**a**); SOX2 (**b**); Nanog (**c**); and Oct4 (**d**).

The immunofluorescent staining of the cells for markers of pluripotency is shown in Figure 5. The positive immunostaining for stage-specific mouse embryonic antigen (SSEA-1), SOX2, Nanog and Oct4 signified the cells were indeed iPSCs, thus, the cells were appropriate for the subsequent cell-differentiation study.

### 2.4. Cytotoxicity of PS80 PBCA NC and PS80 PBCA NC/HRE-cmvBDNF

**Figure 6:**
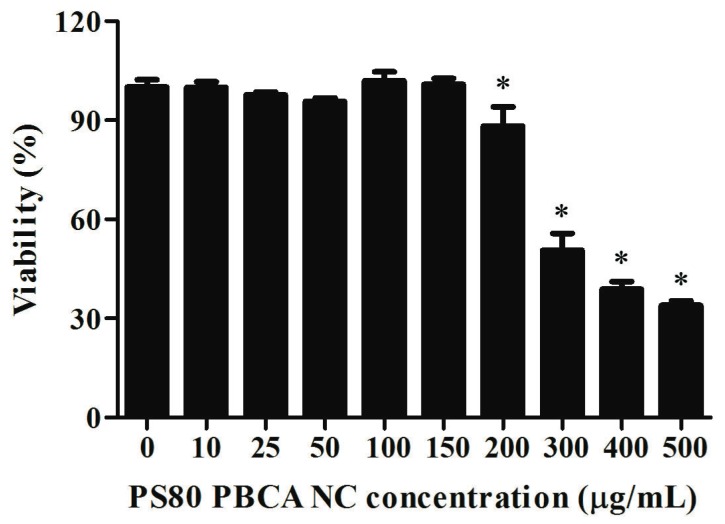
Cytotoxicity of iPSCs to PS80 PBCA NC. Significant cytotoxicity was detected with a PS80 PBCA NC concentration at 200 μg/mL and beyond. *n* = 6; * *p* < 0.001.

**Figure 7:**
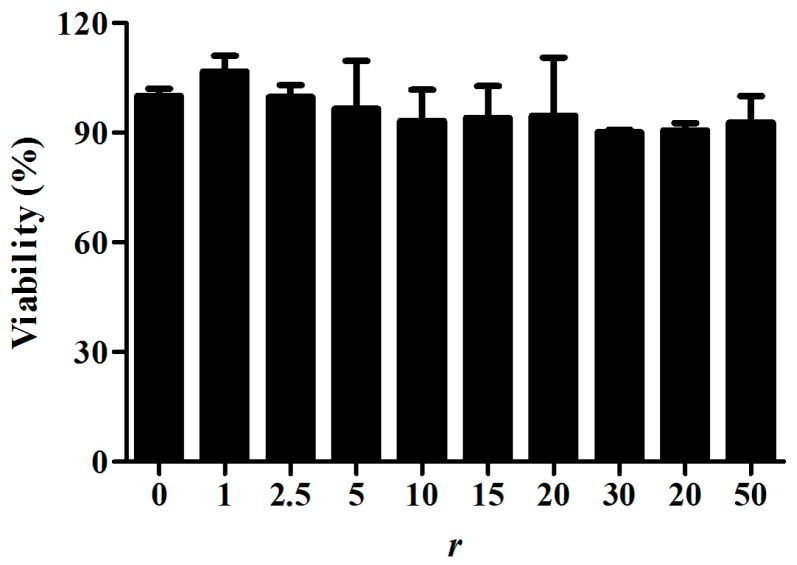
Cytotoxicity of iPSCs to PS80 PBCA NC/HRE-cmvBDNF at different weight ratios. No significant cytotoxicity was observed with a ratio of up to 50:1. *r* is the weight ratio of the PS80 PBCA NC to 2 μg/mL of HRE-cmvBDNF. *n* = 6; *p* > 0.05.

The PS80 PBCA NC and PS80 PBCA NC/HRE-cmvBDNF were tested on iPSCs with the XTT assay for cytotoxicity to determine the appropriate dose for the transfection experiments. A concentration-dependent cytotoxicity profile of the PS80 PBCA NC is shown in Figure 6; the viability of iPSCs was maintained at above 90% from concentrations of 10 to 150 μg/mL, however, further increases in concentration led to a significant reduction in viability. The dose of the HRE-cmvBDNF was chosen at 2 μg/mL, and the cytotoxicity was subsequently determined at different weight ratios (1:1 to 50:1) of PS80 PBCA NC to HRE-cmvBDNF (Figure 7), the maximal PS80 PBCA NC equated to 100 μg/mL, and resulted no significant toxicity between the treated and non-treated cells.

### 2.5. Hypoxia-Responsiveness and Treatment Effect of PS80 PBCA NC/HRE-cmvBDNF on iPSCs

**Figure 8:**
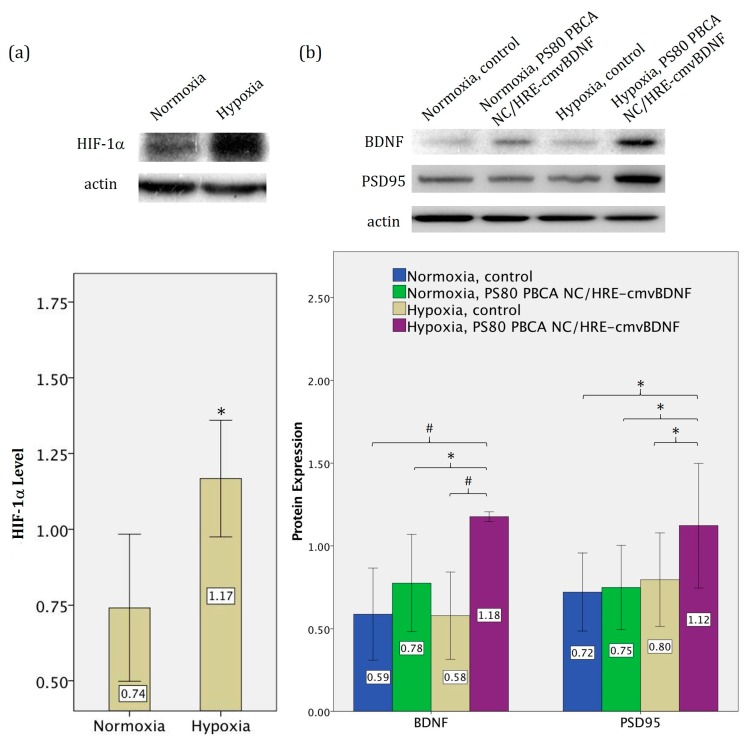
(**a**) The level of HIF-1α in iPSCs exposed to 4 h of hypoxia was significantly higher than in those without hypoxia; and (**b**) BDNF expression was not significantly different in untreated controls with or without prior exposure to hypoxia, and no significant alteration was found in PS80 PBCA NC/HRE-cmvBDNF-treated cells (30 μg/mL:2 μg/mL; r = 15:1) without the hypoxic drive. PS80 PBCA NC/HRE-cmvBDNF-treated cells with prior hypoxia expressed up to 51% more BDNF than those without, and significantly elevated the postsynaptic density protein 95 (PSD95) level. *n* = 4; ^#^ *p* < 0.001; * *p* < 0.05.

The level of HIF-1α with or without hypoxia, and the expression of BDNF and postsynaptic density protein 95 (PSD95) following two days of PS80 PBCA NC/HRE-cmvBDNF transfection as determined by Western blot with semi-quantitative analyses are shown in Figure 8. The exposure of iPSCs to 4 h of hypoxia led to a significant stabilizing effect on the level of HIF-1α, which was 58% higher under hypoxia than in normoxia (*p* < 0.05). In the untreated controls, the basal expression of BDNF was similar in between normoxia and hypoxia. The expression of BDNF in PS80 PBCA NC/HRE-cmvBDNF-treated cells under normoxia was not significantly different to the control; however, the hypoxia-sensing mechanism mediated by the HRE was demonstrated by an up to 100% increase in BDNF level when the treated cells were pre-exposed to hypoxia (*p* < 0.001), and this corresponded to a 56% rise in PSD95 when compared with the respective controls under normoxia.

### 2.6. Treatment Effect of PS80 PBCA NC, HRE-cmvBDNF or PS80 PBCA NC/HRE-cmvBDNF by Immunofluorescent Staining and Western Blotting

**Figure 9:**
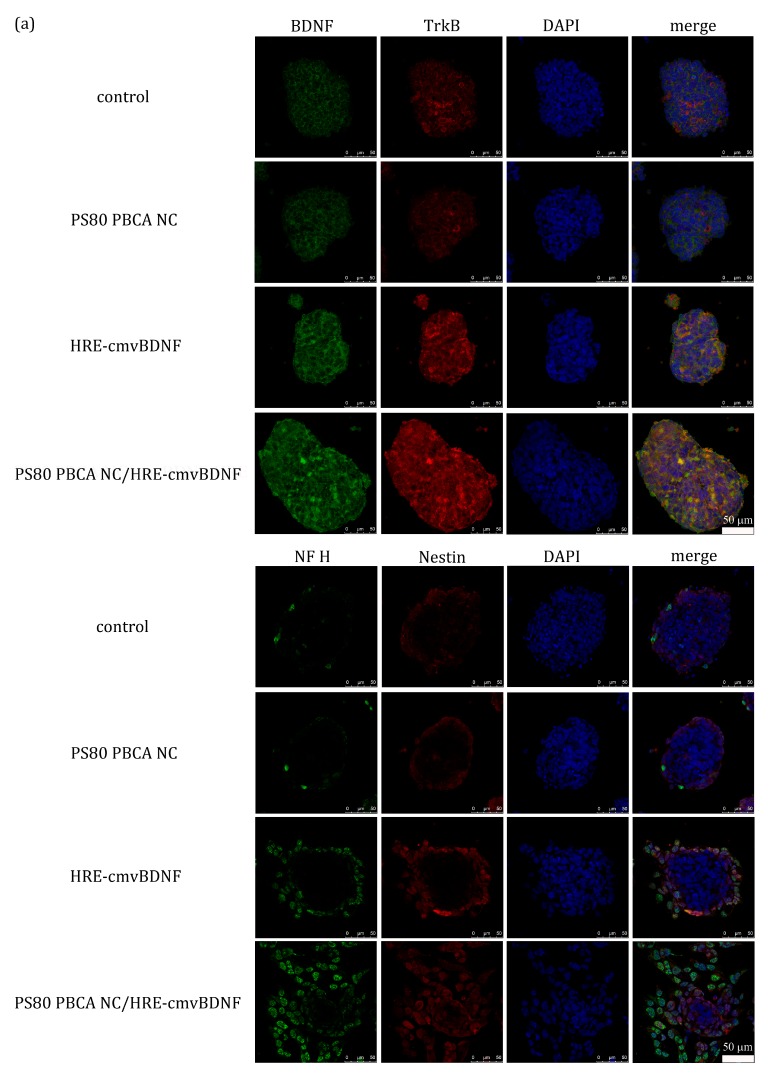

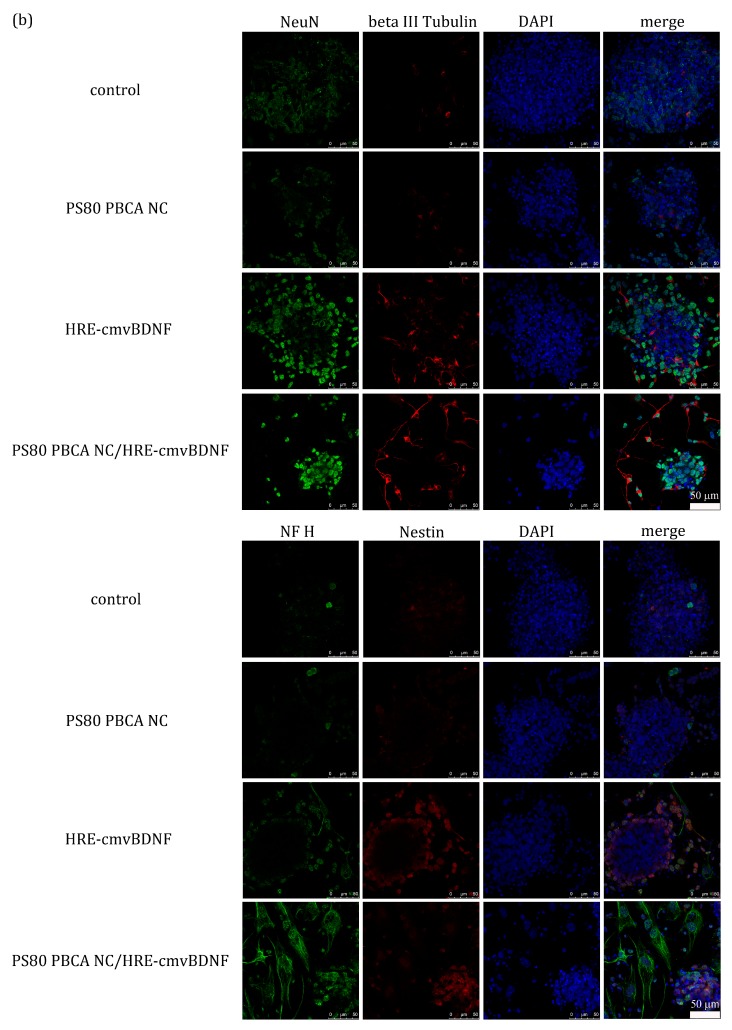

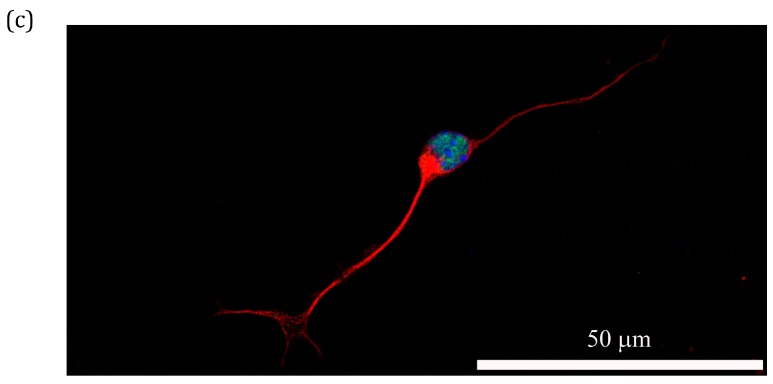
(**a**) Immunofluorescent staining of iPSCs carrying BDNF, tropomysin-related kinase B (TrkB), neurofilament heavy polypeptide (NF H) and Nestin with prior exposure to hypoxia at day 2 after transfection with PS80 PBCA NC (30 μg/mL), HRE-cmvBDNF (2 μg/mL) or PS80 PBCA NC/HRE-cmvBDNF (30 μg/mL:2 μg/mL; r = 15:1). The control and PS80 PBCA NC-treated cells displayed minimal fluorescent intensities for BDNF, TrkB, NF H and Nestin, by contrast the expression levels were higher in those treated with HRE-cmvBDNF and PS80 PBCA NC/HRE-cmvBDNF. The cells of PS80 PBCA NC/HRE-cmvBDNF appeared more loosely packed and spread out, and the expression of NF H and Nestin appeared more homogenous. (**b**) Immunofluorescent staining of iPSCs carrying NF H, Nestin, neuronal nuclei (NeuN) and beta III tubulin at day 7 after transfection with PS80 PBCA NC, HRE-cmvBDNF or PS80 PBCA NC/HRE-cmvBDNF. The fluorescent signals were weak for the control and PS80 PBCA NC-treated cells, neural differentiation was scant and ineffective, by contrast, HRE-cmvBDNF-treated cells showed a stronger signal intensity for all the markers of neural differentiation, and cells of neuron-like morphology were identified. PS80 PBCA NC/HRE-cmvBDNF-treated cells predominantly expressed late marker of neural differentiation such as NF H; the morphological changes were distinctively neuron-like. (**c**) An enlarged view of the PS80 PBCA NC/HRE-cmvBDNF-treated cell tripled stained with NeuN, beta III tubulin and 4′,6-diamidino-2-phenylindole (DAPI) showing a bipolar morphology with an enlarged cell body, elongated axons and dendrites.

The immunofluorescent staining for BDNF, tropomysin-related kinase B (TrkB), neurofilament heavy polypeptide (NF H) and Nestin at two days after transfection with PS80 PBCA NC (30 μg/mL), HRE-cmvBDNF (2 μg/mL) or PS80 PBCA NC/HRE-cmvBDNF (30 μg/mL:2 μg/mL; r = 15:1) in iPSCs pre-exposed to hypoxia is illustrated in Figure 9a. The results showed enhanced expression of BDNF, TrkB, NF H and Nestin by the iPSCs treated with HRE-cmvBDNF and PS80 PBCA NC/HRE-cmvBDNF, by contrast, the expression levels were low and similar in the control and PS80 PBCA NC-treated cells. The iPSCs remained as tightly packed globoid colonies, except in those treated with HRE-cmvBDNF or PS80 PBCA NC/HRE-cmvBDNF where individual cells appeared more loosely packed and dispersed in the periphery, in addition, NF H and Nestin were more homogeneously expressed by PS80 PBCA NC/HRE-cmvBDNF-treated cells.

The immunofluorescent staining for NF H, Nestin, neuronal nuclei (NeuN) and beta III tubulin at seven days after transfection is displayed in Figure 9b. Again, the immunofluorescent intensities for the respective neuronal markers were comparatively weak in the control and PS80 PBCA NC-treated cells; the cells remained as globoid colonies, which consisted of small round to ovoid-shaped cells of similar morphology to undifferentiated iPSCs, and only very occasional cells of neuron-like morphology on beta III tubulin staining were identified. The HRE-cmvBDNF-treated cells showed an elevated expression of NF H, Nestin and NeuN; the cell morphology was more neuron-like, which consisted of a large cell body with elongated axon and fibrillary dendrites, however, the inductiveness of neural differentiation appeared inefficient as evidenced by a large proportion of undifferentiated cells in the background. The PS80 PBCA NC/HRE-cmvBDNF-treated cells exhibited a predominant expression of late neuronal markers such as NF H; neuron-like features were well defined, which occurred in a background of fewer undifferentiated cells.

**Figure 10:**
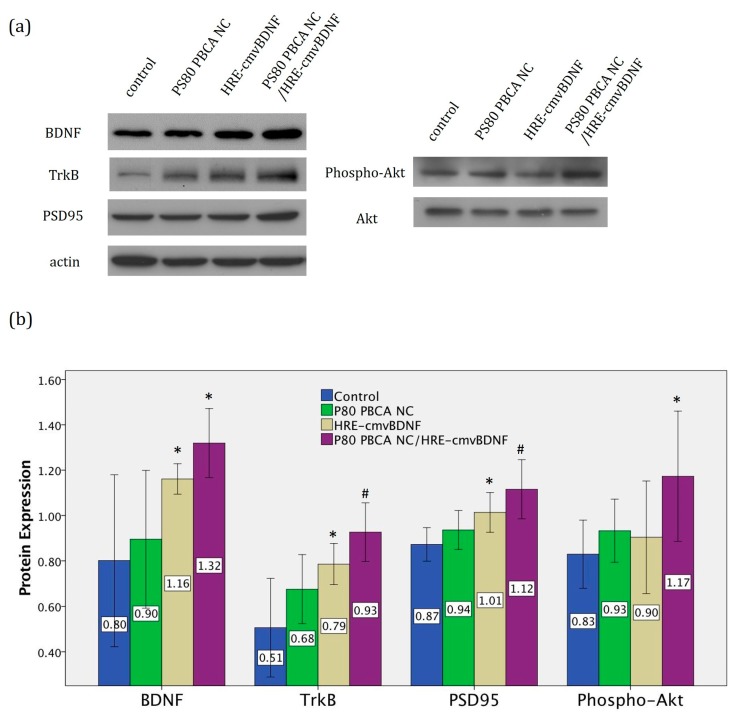
(**a**) Western blot analysis of BDNF, TrkB, PSD95 and Phospho-Akt after two days of transfection; the PS80 PBCA NC/HRE-cmvBDNF-treated cells displayed the strongest band intensities for all the proteins specified above. (**b**) Semi-quantitative analysis of Western blot; the plot shows a progressive rise in the expression level of the respective proteins for the HRE-cmvBDNF and PS80 PBCA NC/HRE-cmvBDNF-treated groups, being most significant in the latter. PBCA NC (30 μg/mL), HRE-cmvBDNF (2 μg/mL) and PBCA NC/HRE-cmvBDNF (30 μg/mL:2 μg/mL; r = 15:1). *n* = 4; ^#^ *p* < 0.001; * *p* < 0.05, compared to control.

The Western blot analyses for BDNF, TrkB and Phospho-Akt at day 2 after transfection are shown in Figure 10. The PS80 PBCA NC/HRE-cmvBDNF-treated group (30 μg/mL:2 μg/mL; r = 15:1) showed a significant increase in the expression of BDNF and its cognante receptor TrkB (*p* < 0.001), A significant elevation of phospho-Akt level (*p* = 0.025) was found correspondingly, which signified activation of the PI3/Akt pathway, and neural differentiation was indicated by the highest expression of the neuron-specific protein PSD95 (*p* < 0.001). The expression profile obtained from the HRE-cmvBDNF-treated group (2 μg/mL) was of intermediate quantity, whereas no significant difference was demonstrated in between the control and PS80 PBCA NC (30 μg/mL) groups.

## 3. Discussion

The surface physicochemical properties such as size, shape and charge of the NCs dictate its interaction with the biological system. The desired properties of PBCA NC are adjustable depending on the polymerization method, pH of the aqueous medium and the selection of surfactants or stabilizers [48,49,50]. Surface decoration with the non-ionic surfactant PS80 does not significantly alter the size or zeta potential of the PBCA NC [51]. The size of HRE-cmvBDNF-adsorbed PBCA NC falls within the range of 20 to 100 nm, which is ideal for systemic administration, because within this size-range renal excretion and reticuloendothelial clearance are minimized [52,53], thus circulation half-life is prolonged to enable greater amounts of therapeutics reaching the CNS. The negative zeta potential of PS80 PBCA NC arises from the adsorption of anions within the aqueous polymerization medium, adding HRE-cmvBDNF has a stabilizing effect, because the greater the NC’s zeta potential, regardless of being positive or negative, will serve to prevent aggregation of the particles in suspension by electrostatic repulsion [54]. A negative zeta potential avoids phagocytosis by the reticuloendothelial system and non-specific cytotoxicity; however, the cellular internalization and adsorption of *p*DNA onto the NC may be less efficient when compared with cationic NCs [55,56,57]. Although the negative charge associated with the PS80 PBCA NC/HRE-cmvBDNF may appear disadvantageous, it may serve to limit non-specific cellular uptake to avoid high basal BDNF expression and theoretically makes the hypoxia-sensing response more site-specific and stimulus-driven; this effect was demonstrated by an elevated BDNF expression in PS80 PBCA NC/HRE-cmvBDNF-treated cells only with prior exposure to hypoxia.

Spherical NCs constitute the most basic and common geometry of nanoscale-drug delivery system [58], they are more readily internalized than non-spherical ones, but it possess hydrodynamic features that results in a shorter circulation half-life, and are less able to escape from phagocytic clearance [59]. The observed change in surface texture suggests adsorption of the *p*DNAs onto the NC, and the interactive forces are presumed to be non-covalent in nature with desorption as the main release mechanism. This mode of delivery has been shown to partially protect *p*DNAs from nuclease degradation by electrophoretic mobility study [60], furthermore, desorption or unloading of the *p*DNA for transcription may be better facilitated by the weak interaction between *p*DNA and anionic PS80 PBCA NC, however, the immunogenicity to these surface-bound *p*DNAs will need to be investigated further.

PS80-coated PBCA NCs have been shown to be less toxic than those made with other surfactants [61]. In our study, coating of PBCA NC with PS80 extended the tolerance of iPSCs to 150 μg/mL of PBCA NC; which is a 15-fold increase in maximal safe concentration when compared with a previous report using uncoated PBCA NC for NT-3 gene delivery [36]. A major drawback of PBCA NC is the burst-release profile that may result in losses of up to 40%–70% of its cargo [62], and, since the transfection efficiency of the vector is proportional to the loading percentage [63], the decline in efficacy may be much pronounced when the cargo is adsorbed on the surface of the NC as is the case of PS80 PBCA NC/HRE-cmvBDNF.

Multiple signaling pathways have been implicated in the neural differentiation of stem cells [64]. The mammalian target of rapamycin (mTOR) pathway down stream of PI3K/Akt appears to play a role in neural differentiation of mouse embryonic stem cells [65]. Our result showed that the enhanced expression of BDNF coupled the recruitment of TrkB receptors, and activation of the PI3K/Akt signaling pathway might also be responsible for the neural differentiation of iPSCs. The elevated level of HIF-1α in iPSCs under hypoxia signified an intact hypoxia-sensing mechanism, and that hypoxia could indeed augment BDNF expression from the delivered HRE-cmvBDNF gene. Therefore, the inclusion of a HRE in the gene construct offers a hypoxia-mediated trigger for BDNF expression, and may potentially be used to target CNS insults where tissue hypoxia occur.

The inherent capability of iPSCs to differentiate into neurons by the “neural default model” of stem cell differentiation have been described [66,67], but it appeared very inefficient under our study condition and time frame as seen in the non-treated control and the PS80 PBCA NC-treated groups. However, collective analyses from the cell morphology, immunofluorescence and Western blot demonstrated that the extent of neural inductiveness on iPSCs by the PS80 PBCA NC/HRE-cmvBDNF was superior to HRE-cmvBDNF alone. The observed differences can be explained by the stability of *p*DNA, ease of cell internalization, and intracellular degradation. Firstly, the association of *p*DNAs with low-molecular weight polycations or basic polypeptides in the biologic medium may result in uncontrolled aggregation and precipitation [68], by contrast, *p*DNAs condensed on NCs are stable and can resist aggregation. Secondly, the stability gained may protect the *p*DNA from nuclease degradation in the extra- and intracellular compartments. Thirdly, NCs facilitate a reduction in the net charge of *p*DNAs, thus enabling a greater extent of interaction with the anionic proteoglycans on the cell membrane for endocytosis [69,70]. Lastly, PBCA NC is known to be stable under acidic conditions such as those in the lysosome; they are only degraded once it has escaped into the cytosol, therefore, this pH-dependent release profile is advantageous for the delivery of therapeutics [27,71].

We have demonstrated in this study the feasibility and neural differentiation capability of iPSCs treated by a hypoxia-sensing non-viral vector comprised of PS80 PBCA NC and HRE-cmvBDNF. The nano-formulation may be best suited for targeted BDNF-induced neuroregeneration or neuroprotection where perturbation in CNS oxygenation lies such as those seen in ischemic or hemorrhagic strokes, however, the modes of administration, pharmacokinetics and toxicity profile will need to be evaluated in future in vivo studies, and, finally, the treated iPSCs may be transplanted as a cell-based BDNF delivery strategy [72,73], where the overexpressed BDNF not only may act on the donor stem cells, but also promote neural differentiation of resident stem cells.

## 4. Materials and Methods

### 4.1. Construction of HRE-cmvBDNF Plasmid DNA

The functional domains of BDNF amino acid 155–273 was cloned and sequenced. The DNA fragment was amplified by polymerase chain reaction with the restriction enzymes BamHI (Fermentas, Vilnius, Lithuania) and Hind III (Fermentas) attached to the recognition sites of 5’-GGATCCCACTCCGACCCCGCCCG-3’ and 5’-AAGCTTTCTTCCCCTTTTTAATGGTCAGT-3’ respectively. The SV40 promoter in pSV-β-galactosidase control vector (Promega, Madison, WI, USA) was replaced with the CMV promoter from the pGEM base vector (Promega), then digested with BsaA I (New England BioLabs, Ipswich, MA, USA) and Ava I (New England BioLabs), and cloned with the amplified BDNF DNA fragment. HREs (P18 × 3) with the P18 sequence TGTCACGTCCTGCACGAC were inserted into the Sal I site. The constructed HRE-cmvBDNF *p*DNA was subsequently transformed to *Escherichia coli* JM 109 cells (ECOS^™^ 9–5; Yeastern Biotech, Taipei, Taiwan). Quick plasmid preparation (Mini Plus^™^ Plamid DNA Extraction System, Viogene, New Taipei City, Taiwan) and enzyme digestions selected the positive clones. The correct clones were verified by DNA sequence determination.

### 4.2. Preparation of PS80-Coated PBCA NC and PS80-Coated PBCA NC/HRE-cmvBDNF

PS80 PBCA NC was synthesized by the emulsion polymerization technique reported previously with minor modifications [74,75,76]. Concisely, 1% (*v*/*v*) butylcyanoacrylate monomers (BCA, Sicomet, Sichel Werk, Hanover, Germany) were added drop by drop into 0.01 N HCl acidic polymerization medium containing 1% (*w*/*v*) dextran 70,000 (Sigma, St. Louis, MO, USA) and 0.1% (*v*/*v*) polysorbate 80 (PS80) as a stabilizer at pH 2.4 under 400 rpm and 25 °C for 3 h. Then, 0.1 N NaOH was mixed with PBCA NC suspension to terminate polymerization. The suspension was then centrifuged at 5100× *g* for 10 min. The larger polymer aggregates were removed from the nanoparticles suspension by filtration through a 0.45 μm filtration unit.

The PS80 PBCA NC/HRE-cmvBDNF was made by mixing the fully formed PS80 PBCA NC with HRE-cmvBDNF in Tris buffer or DPBS; the exact proportion of each components are described in subsequent sections where PS80 PBCA NC/HRE-cmvBDNF was used.

### 4.3. Characterization of NC

#### 4.3.1. Particle Size and Zeta Potential

0.2 mg/mL of the synthesized PS80 PBCA NC and PS80 PBCA NC/HRE-cmvBDNF at pH 7.4 DPBS buffer and 25 °C were used for characterization of the cumulant Z-average diameter (*D*av), zeta potential (ζ) and polydispersity index (PDI) by a Zetasizer Nano ZS90 (Malvern, Worcestershire, UK) with a photo correlation spectroscopy.

#### 4.3.2. Morphology of NC

The NC suspension of 0.2 mg/mL was loaded on a carbon-coated 200-mesh copper grid for 2 min. The samples were vacuum-dried and sputter-coated with platinum at 2 kV for 90 s. The NC surface structure was obtained by a field emission scanning electron microscope (FE-SEM, SU-8220, Hitachi, Tokyo, Japan). In addition, the samples were loaded on a copper grid and stained with 2% (*w*/*v*) phosphotungstic acid solution for 2 min. The transmission electron microscope (TEM, H-7500, Hitachi) was used to obtain images of the particle structure.

#### 4.3.3. pDNA Adsorption Efficiency of PS80 PBCA NC

The different weight ratios (1:1 to 50:1) of PS80 PBCA NC to HRE-cmvBDNF made to 100 μL with Tris buffer (pH = 7.4) were prepared; the dose of HRE-cmvBDNF was held at 2 μg/mL, which was taken in reference to the high-end of the recommended *p*DNA concentration range of 0.4–2 μg/mL for the preparation of transfection liposome-DNA complexes [77]. The samples were spun down at 13,200 rpm for 15 min after 1 h of incubation at room temperature. The total amount of *p*DNA (A) was calculated according to the doses of *p*DNA used; the unadsorbed free *p*DNA (B) in the supernatant was quantified by an ND-1000 spectrophotometer (Nano Drop, Thermo Fisher Scientific, Waltham, MA, USA) at 260 nm wavelength. The *p*DNA adsorption efficiency (*AE*) of PS80 PBCA NC was calculated by:

*AE* (%) = (A − B)/A × 100

The supernatant *p*DNA was separated by 0.8% agarose gel (Sigma) electrophoresis, and images were obtained with a ultraviolet (UV) transilluminator (UVltec, Cambridge, UK).

### 4.4. Cell Culture

Mouse iPSCs (SC201A-iPSC, passage 3, System Biosciences, Mountain View, CA, USA) were maintained in ESGRO complete plus clonal grade medium. The cells were passaged on reaching 80%–90% confluence at a ratio of 1:4 in 75 cm^2^ flasks pre-coated with 0.1% gelatin. iPSCs of passages 11–13 were used for all experiments. The iPSCs were checked for SSEA-1, SOX2, Nanog and Oct4 expression by immunocytochemistry with the respective antibodies: mouse monoclonal anti-SSEA1-PE antibody (1:100, Millipore, Temecula, CA, USA), rabbit polyclonal anti-SOX2 (1:1000, Abcam, Cambridge, MA, USA), rabbit polyclonal anti-Nanog (1:700, Abcam) and rabbit polyclonal anti-Oct4 (1:1000, Abcam) at 4 °C overnight and fluorescein isothiocyanate-conjugated secondary antibody (1:100, Millipore) at room temperature for 1 h, images were captured by a fluorescence microscope (Eclipse E600, Nikon, Tokyo, Japan).

### 4.5. Cytotoxicity of NC

The cytotoxicity of iPSCs to PS80 PBCA NC and PS80 PBCA NC/HRE-cmvBDNF was estimated by the XTT assay (cell proliferation kit, Biological Industries, Beit Haemek, Israel). iPSCs were seeded into a gelatin-coated 96-well plate at a density of 5000 cells/well and incubated for 8 h. One hundred microliter of growth medium per well containing different concentrations of PS80 PBCA NC or PS80 PBCA NC/HRE-cmvBDNF were added, and incubated for 4 h followed by the XTT assay. The absorption was measured by an EnSpire Multimode Plate Reader (Perkin Elmer Inc., Waltham, MA, USA) at a wavelength of 490 nm.

### 4.6. In Vitro Transfection and Protein Expression with Pre-Exposure to Hypoxia by Immunofluorescent Staining

PS80 PBCA NC/HRE-cmvBDNF was used to determine the transfection efficiency. iPSCs were seeded into gelatin-coated 24-well plates at a density of 2 × 10^5^ cells/well. The cell cultures were incubated under hypoxia (5% CO_2_, 1% O_2_ and 95% relative humidity) at 37 °C for 4 h before transfection. A ratio 15:1 of PS80 PBCA to HRE-cmvBDNF was taken from *p*DNA adsorption efficiency and cytotoxicity experiments; PS80 PBCA NC/HRE-cmvBDNF was prepared by adding 2 μg *p*DNA and 30 μg PBCA NC in DPBS buffer for 1 h, and made to 1 mL with ESGRO Complete Plus clonal grade medium. The iPSCs were incubated with the PS80 PBCA NC/HRE-cmvBDNF for 4 h; the medium containing PS80 PBCA NC/HRE-cmvBDNF was then replaced with fresh ESGRO culture medium and returned to normal incubation conditions for two days or seven days. The cells were fixed with 4% formaldehyde solution for 10 min and stained with the primary antibody against BDNF (1:1000, Abcam), TrkB (1:200, Abcam), neurofilament heavy (NF H; 1:200, Abcam), Nestin (1:200, Abcam), Neuronal nuclei (NeuN; 1:100, Abcam) and beta III tubulin (1:200, Abcam) at 4 °C overnight. The sample were then incubated with secondary antibodies conjugated to Alexa Fluor^®^ 488 (1:1000, Abcam) or Alexa Fluor^®^ 594 (1:1000, Abcam) at room temperature for 1 h. Cell nuclei were stained with 4′,6-diamidino-2-phenylindole (DAPI). The images were visualized and captured using a confocal laser scanning microscope.

### 4.7. Western Blot

#### 4.7.1. Hypoxia-Responsiveness and Treatment Effect of PS80 PBCA NC/HRE-cmvBDNF on iPSCs

The hypoxia-responsiveness was determined by the level of HIF-1α expression following 4 h of hypoxia. The treatment effect of PS80 PBCA NC/HRE-cmvBDNF was assessed after two days of transfection with or without pre-exposure to hypoxia. iPSCs were seeded at a density of 1 × 10^5^ cells/cm^2^ into 10-cm dishes and incubated under normoxic (5% CO_2_, 21% O_2_ and 95% relative humidity) or hypoxic condition for 4 h before transfection. PS80 PBCA NC /HRE-cmvBDNF was prepared by adding 30 μg *p*DNA and 450 μg PS80 PBCA NC in DPBS buffer for 1 h and made to 15 mL with ESGRO Complete Plus clonal grade medium. The iPSCs were incubated with the PS80 PBCA NC/HRE-cmvBDNF for 4 h, the medium containing PS80 PBCA NC/HRE-cmvBDNF was then replaced with fresh ESGRO culture medium and incubated under normal conditions for two days. The cells were lysed in a 1 × RIPA buffer (20 mM Tris buffer, pH 7.5, 150 mM NaCl, 1% NP-40, 1% sodium deoxylcholate) for protein extraction. The soluble protein concentration was estimated using a bicinchoninic acid (BCA) protein assay kit (Santa Cruz Biotechnology, Santa Cruz, CA, USA). Fifty microgram of protein was resolved by a 10% sodium dodecyl sulfate-polyacrylamide gel electrophoresis (SDS-PAGE) and transferred to a polyvinylidene fluoride (PVDF) membrane. The membrane was blocked with 5% non-fat milk and hybridized with the primary antibody against HIF-1α (1:500, Novus Biologicals, Littleton, CO, USA), BDNF (1:1000, Santa Cruz Biotechnology, Santa Cruz, CA, USA), PSD95 (1:1000, Abcam) or β-actin (1:10,000, Sigma-Aldrich, St. Louis, MO, USA) overnight at 4 °C. Then the membranes were washed and incubated with a specific secondary antibody for 1 h at room temperature and a sensitive chemiluminescent horseradish peroxidase substrate (Amersham ECL Prime, GE Health Care Life Sciences, Uppsala, Sweden) was used to detect protein signals. Autoradiographic signals were detected by X-ray film (Fuji Medical X-ray Film, Tokyo, Japan). The signal intensity was quantified by Gene tools analysis software (SYNGEN, Cambridge, UK).

#### 4.7.2. Treatment Effect of PS80 PBCA NC, HRE-cmvBDNF or PS80 PBCA NC/HRE-cmvBDNF on iPSCs Pre-Exposed to Hypoxia

iPSCs were seeded at a density of 1 × 10^5^ cells/cm^2^ into 10-cm dishes and incubated under hypoxia for 4 h before transfection. PS80 PBCA NC /HRE-cmvBDNF was prepared as in Section 4.7.1. The iPSCs were incubated with the PS80 PBCA NC, HRE-cmvBDNF or PS80 PBCA NC/HRE-cmvBDNF for 4 h, the medium was then replaced with fresh ESGRO culture medium and incubated under normal conditions for two days. Fifty micrograms of proteins were analyzed by Western blotting. The primary antibodies used in this experiment were BDNF (1:1000, Santa Cruz Biotechnology, Santa Cruz, CA, USA), TrkB (1:1000, Abcam), PSD95 (1:1000, Abcam), Akt (1:4000, Cell Signaling, Danvers, MA, USA), Phospho-Akt (1:1000, Cell Signaling) and β-actin (1:10,000, Sigma-Aldrich, St. Louis, MO, USA).

### 4.8. Statistical Analysis

The numeric variables were expressed as mean ± standard deviation. All statistical analyses were performed by using GraphPad Prism^®^ 5 software (La Jolla, CA, USA). The data were analyzed with a one-way analysis of variance (ANOVA) and Bonferroni pose hoc test. A *p* value of 0.05 or less indicates a significant statistical difference.

## 5. Conclusions

The non-viral nano-scaled polymeric gene delivery platform consisting of PS80 PBCA NC/HRE-cmvBDNF was constructed and the capability for neural differentiation of iPSCs driven by hypoxia was evaluated. The study demonstrated that PS80 PBCA NC served as an efficient delivery platform for BDNF gene coupled to the HRE in hypoxia-sensitive cells, and the neural inductiveness by PS80 PBCA NC/HRE-cmvBDNF was indeed superior to HRE-cmvBDNF alone in vitro. Furthermore, iPSCs were capable of neural differentiation via activation of the PI3/Akt pathway by the forced expression of BDNF.

## Figures and Tables

**Table 1:** Average diameter (*D*av), zeta potential and polydispersity index (PDI) of the nanocarriers.

Sample	*D*av	Zeta Potential	PDI
	(nm)	(mV)	
PS80 PBCA NC	88.3 ± 1.5	−5.3 ± 0.7	0.04 ± 0.02
PS80 PBCA NC/HRE-cmvBDNF	92.6 ± 0.9	−14.1 ± 1.1	0.10 ± 0.02

*n* = 6; *p* < 0.001.
